# Supplementary material for: Analysis of Potential Genes and Economic Parameters Associated with Growth and Heat Tolerance in Sheep (Ovis aries)
Source: Animals (Basel). 2023 Jan 19;13(3):353. doi: 10.3390/ani13030353 (PMC9913162; doi:10.3390/ani13030353)
Supplement: Supplementary file 1 [file animals-13-00353-s001.zip › animals-2054219-supplementary.pdf]

Supplementary Materials

```
LC717976.1      GCTGGGTCTTCATTGCTGTGTTCAGGCTTTCTCTAGTTGGGGCAAGCGAGGGCTATTCTC 60
B               GCTGGGTCTTCATTGCTGTGTTCAGGCTTTCTCTAGTTGGGGCAAGCGAGGGCTATTCTC 60
AD              GCTGGGTCTTCATTGCTGTGTTCAGGCTTTCTCTAGTTGGGGCAAGCGAGGGCTATTCTC 60
                ************************************************************

LC717976.1      TAGTTGCGATGTGCAGCCTTCTGCAGGTGGCTTCTCTTGTTGCAGAGCCGGGGCTCTGGG 120
B               TAGTTGCGATGTGCAGCCTTCTGCAGGTGGCTTCTCTTGTTGCAGAGCCGGGGCTCTGGG 120
AD              TAGTTGCGATGTGCAGCCTTCTGCAGGTGGCTTCTCTTGTTGCAGAGCCGGGGCTCTGGG 120
                ************************************************************

LC717976.1      TGCACAGGCTTCAGTTGTTGTGGCTTGAAGCTCTAGAGCACAGGCTCAGTGGTCGTGGCG 180
B               TGCACAGGCTTCAGTTGTTGTGGCTTGAAGCTCTAGAGCACAGGCTCAGTGGTCGTGGCG 180
AD              TGCACAGGCTTCAGTTGTTGTGGCTTGAAGCTCTAGAGCACAGGCTCAGTGGTCGTGGCG 180
                ************************************************************

LC717976.1      CACAGGCTTACTCCACGGCATGTGGGATCTTCCCTGGCCAGGGAGCGAACCCGTGTCCCC 240
B               CACAGGCTTACTCCATGGCATGTGGGATCTTCCCTGGCCAGGGAGCGAACCCGTGTCCCC 240
AD              CACAGGCTTACTCCACGGCATGTGGGATCTTCCCTGGCCAGGGAGCGAACCCGTGTCCCC 240
                *************** ********************************************

LC717976.1      TGCGTTGCAAGGCGGCCTCTTAACCACTGGCCACCAGGGAAGCCCCAAGATGCCAAGGCT 300
B               TGCGTTGCAAGGCGGCCTCTTAACCACTGGCCACCAGGGAAGCCCCAAGATGCCAAGGCT 300
AD              TGCGTTGCAAGGCGGCCTCTTAACCACTGGCCACCAGGGAAGCCCCAAGATGCCAAGGCT 300
                ************************************************************

LC717976.1      TTTTACTTCTGGTTCTTACCGTCTGGTTAATACAATTCCTTCATCAGCCAGTCA 354
B               TTTTACTTCTGGTTCTTACCGTCTGGTTAATACAATTCCTTCATCAGCCAGTCA 354
AD              TTTTACTTCTGGTTCTTACCGTCTGGTTAATACAATTCCTTCATCAGCCAGTCA 354
                ******************************************************
```

Figure S1. Representative DNA sequence alignment for a segment of CDS of *CAST* gene (354-bp) among Barki and Aboudeleik lambs and reference sequence available in the GenBank gb|LC717976.1|. B= Barki and AD= Aboudeleik. The asterisks represent the similarity.

```
AH014693.2      ACTGGTTTGGACTTCATCCCTGGGCTCCACCCTCTCCTGAGTTTGTCCAAGATGGACCAG 60
B               ACTGGTTTGGACTTCATCCCTGGGCTCCACCCTCTCCTGAGTTTGTCCAAGATGGACCAG 60
AD              ACTGGTTTGGACTTCATCCCTGGGCTCCACCCTCTCCTGAGTTTGTCCAAGATGGACCAG 60
                ************************************************************

AH014693.2      ACATTGGCAATCTACCAACAGATCCTCGCCAGTCTGCCTTCCAGAAATGTGATCCAAATA 120
B               ACATTGGCGATCTACCAACAGATCCTCGCCAGTCTGCCTTCCAGAAATGTGATCCAAATA 120
AD              ACATTGGCAATCTACCAACAGATCCTCGACAGTCTGCCTTCCAGAAATGTGATCCAAATA 120
                ******** ******************* *******************************

AH014693.2      TCTAATGACCTGGAGAACCTCCGGGACCTTCTCCACCTGCTGGCCGCCTCCAAGAGCTGC 180
B               TCTAATGACCTGGAGAACCTCCGGGACCTTCTCCACCTGCTGGCCGCCTCCAAGAGCTGC 180
AD              TCTAATGACCTGGAGAACCTCCGGGACCTTCTCCACCTGCTGGCCGCCTCCAAGAGCTGC 180
                ************************************************************

AH014693.2      CCCTTGCCGCAGGTCAGGGCCCTGGAGAGCTTGGAGAGCCTGGGCGTCGTCCTGGAAGCC 240
B               CCCTTGCCGCAGGTCAGGGCCCTGGAGAGCTTGGAGAGCCTGGGCGTCGTCCTGGAAGCC 240
AD              CCCTTACCGCAGGTCAGGGCCCTGGAGAGCTTGGAGAGCCTGGGCGTCGTCCTGGAAGCC 240
                ***** ******************************************************

AH014693.2      TCCCTCTACTCCACCGAGGTGGTGGCCCTGAGCCGGCTACAGGGGTCTCTACAGGACATG 300
B               TCCCTCTACTCCACCGAGGTGGTGGCCCTGAGCCGGCTACAGGGGTCTCTACAGGACATG 300
AD              TCCCTCTACTCCACCGAGGTGGTGGCCCTGAGCCGGCTACAGGGGTCTCTACAGGACATG 300
                ************************************************************

AH014693.2      TTGCGGCAGCTGGACCTCAGCCCTGGGTGCTGAAGCCTTGAAGGCCTCTCTTCCCAAAGT 360
B               TTGCGGCAGCTGGACCTCAGCCCTGGGTGCTGAAGCCTTGAAGGCCTCTCTTCCCAAAGT 360
AD              TTGCAGCAGCTGGACCTCAGCCCTGGGTGCTGAAGCCTTGAAGGCCTCTCTTCCCAAAGT 360
                **** *******************************************************

AH014693.2      CCAGGGAAGAAACCTGAGCTTCTGGCTGTCTGCAGAAGAGAGCCTATGTGGGCATCCTTT 420
B               CCAGGGAAGAAACCTGAGCTTCTGGCTGTCTGCAGAAGAGAGCCTATGTGGGCATCCTTT 420
AD              CCAGGGAAGAAACCTGAGCTTCTGGCTGTCTGCAGAAGAGAGCCTATGTGGGCATCCTTT 420
                ************************************************************

AH014693.2      ATGCAGGCCAGC       432
B               ATGCAGGCCAGC       432
AD              ATGCAGGCCAGC       432
                ************
```

Figure S2. Representative DNA sequence alignment for a segment of CDS of *LEP* gene (432-bp) among Barki and Aboudeleik lambs and reference sequence available in the GenBank gb|AH014693.2|. B= Barki and AD= Aboudeleik. The asterisks represent the similarity.

```
XM_027958720.2      ATGGGAGCTGTTTCTCCCTCTTGCAGTGGAGAGAAGAGTCACGCTGACTGTGTCCTCATT      60
B                   ATGGGAGCTGTTTCTCCCTCTTGCAGTGGAGAGAAGAGTCACGCTGACTGTGTCCTCATT      60
AD                  ATGGGAGCTGTTTCTCCCTCTTGCAGTGGAGAGAAGAGTCACGATGACTGTGTCCTCATT      60
                    ******************************************* ****************

XM_027958720.2      TACAAATCAGAGACCGAGGGGACAGAGACCACACTCCAGACACGGACTTCCTCGGATTC      120
B                   TACAAATCAGAGACCGAGGGGACAGAGACCACGCTCCAGACACGGACTTCCTCGGATTC      120
AD                  TACAAATCAGAGACCGAGGGGACAGAGACCACACTCCAGACACGGACTTCCTCGGATTC      120
                    ******************************** ***************************

XM_027958720.2      CCCACCAAGATGCTAACAGTGAAGAGGCTGGGAGGAAGCAACGCGTGTTACCGCAGTGAC      180
B                   CCCACCAAGATGCTAACAGTGAAGAGGCTGGGAGGAAGCAACGCGTGTTACTGCAGTGAC      180
AD                  CCCACCAAGATGCTAACAGTGAAGAGGCTGGGAGGAAGCAACGCGTGTTACCGCAGTGAC      180
                    *************************************************** ********

XM_027958720.2      CAGCTGGAGAAGATGGCCTTTTTCCAGTGCATGGAGGAGGTGGAGAAGTGCTTTCTGGAA      240
B                   CAGCTGGAGAAGATGGCCTTTTTCCAGTGCATGGAGGAGGTGGAGAAGTGCTTTCTGGAA      240
AD                  CAGCTGGAGAAGATGGCCTTTTTCCAGTGCATGGAGGAGGTGGAGAAGTGCTTTCTGGAA      240
                    ************************************************************

XM_027958720.2      GAAAGTTCCGGTGAACAAGATTCGAGATCTGGAAAGAACCAGGCAAAGGAGCAAGCGTGG      300
B                   GAAAGTTCCGGTGAACAAGATTCGAGATCTGGAAAGAACCAGGCAAAGGAGCAAGCGTGG      300
AD                  GAAAGTTCTGGTGAACAAGATTCGAGATCTGGAAAGAACCAGGCAAAGGAGCAAGCGTGG      300
                    ******** ***************************************************

XM_027958720.2      TCACTCCCTGGCCTGCAGGAGACAGCACCCGCCGCCGGTGAAAGGACAGCGGCCCCCGCA      360
B                   TCACTCCCTGGCCTGCAGGAGACAGCACCCGCCGCCGGTGAAAGGACAGCGGCCCCCGCA      360
AD                  TCACTCCCTGGCCTGCAGGAGACAGCACCCGCCGCCGGTGAAAGGACAGCGGCCCCCGCA      360
                    ************************************************************

XM_027958720.2      GCCGACGCCCTGGCACCGCCTGCGCCCTTTGACCACCGCATCGTGACGGCCAGGCA 416
B                   GCCGACGCCCTGGCACCGCCTGCGCCCTTTGACCACCGCATCGTGACGGCCAGGCA 416
AD                  GCCGACGCCCTGGCACCGCCTGCGCCCTTTGACCACCGCATCGTGACGGCCAGGCA 416
                    ********************************************************
```

Figure S3. Representative DNA sequence alignment for a segment of CDS of *MYLK4* gene (416-bp) among Barki and Aboudeleik lambs and reference sequence available in the GenBank gb|XM_027958720.2|. B= Barki and AD= Aboudeleik. The asterisks represent the similarity.

```
XM_042249577.1      GGTAACATTCACCAAGCGGAAGTTCGGGCTGATGAAGAAGGCCTACGAGCTGAGCGTGCT      60
B                   GGTAACATTCACCAAGCGGAAGTTCGGGCTGATGAAGAAGGCCTACGAGCTGAGTGTGCT      60
AD                  GGTAACATTCACCAAGCGGAAGTTCGGGCTGATGAAGAAGGCCTACGAGCTGAGCGTGCT      60
                    ****************************************************** *****

XM_042249577.1      CTGCGACTGCGAGATCGCCCTCATCATCTTCAACAGCTCCAACCGCCTCTTCCAGTACGC      120
B                   CTGCGACTGCGAGATCGCCCTCATCATCTTCAACAGCTCCAACCGCCTCTTCCAGTACGC      120
AD                  CTGCGACTGCGAGATCGCCCTCATCATCTTCAACAGCTCCAACCGCCTCTTCCAGTACGC      120
                    ************************************************************

XM_042249577.1      CAGCACCGACATGGACCGCGTGCTTCTGAAGTACACGGAGTACAGCGAGCCTCACGAGAG      180
B                   CAGCACCGACATGGACCGCGTGCTTCTGAAGTACACGGAGTACAGCGAGCCTCACGAGAG      180
AD                  CAGCACCGACATGGACCGCGTGCTTCTGAAGTACACAGAGTACAGCGAGCCTCACGAGAG      180
                    ************************************ ***********************

XM_042249577.1      CCGCACCAACGCCGACATCCTCGAGACACTGAAGCGGAGGGGTGTGGGCCTTGATGGACC      240
B                   CCGCACCAACGCCGACATCCTCGAGACACTGAAGCGGAGGGGTGTGGGCCTTGATGGACC      240
AD                  CCGCACCAACGCCGACATCCTCGAGACACTGAAGCGGAGGGGTGTGGGCCTTGATGGACC      240
                    ************************************************************

XM_042249577.1      CGAGCTGGAGCCAGACGAGGGGCTTGAGGGGCCAGGAGAGAAGCTGCGGAGGCTGGCAGG      300
B                   CGAGCTGGAGCCAGACGAGGGGCTTGAGGGGCCAGGAGAGAAGCTGCGGAGGCTGGCAGG      300
AD                  CGAGCTGGAGCCAGACGAGGGGCTTGAGGGGCCAGGAGAGAAGCTGCGGAGGCTGGGAGG      300
                    ******************************************************** ***

XM_042249577.1      TGATGGGGGTGACCCAGCCTTGCCCCGGCCCCGGCTCTATCCCGCAGCCCCCACTATGCC      360
B                   TGATGGGGGTGACCCAGCCTTGCCCCGGCCCCGGCTCTATCCCGCAGCCCCCACTATGCC      360
AD                  TGATGGGGGTGACCCAGCCTTGCCCCGGCCCCGGCTCTATCCCGCAGCCCCCACTATGCC      360
                    ************************************************************

XM_042249577.1      CAGCCCAGACATGGTATATGGGGCACTGCCCCCACCAGCCTGTGAACCCACTGGACTTGG      420
B                   CAGCCCAGACATGGTATATGGGGCACTGCCCCCACCAGCCTGTGAACCCACTGGACTTGG      420
AD                  CAGCCCAGACATGGTATATGGGGCACTGCCCCCACCAGCCTGTGAACCCACTGGACTTGG      420
                    ************************************************************

XM_042249577.1      GGAG     424
B                   GGAG     424
AD                  GGAG     424
                    ****
```

Figure S4. Representative DNA sequence alignment for a segment of CDS of *MEF2B* gene (424-bp) among Barki and Aboudeleik lambs and reference sequence available in the GenBank gb|XM_042249577.1|. B= Barki and AD= Aboudeleik. The asterisks represent the similarity.

```
NM_001009402.2      TGGGATGCCATCGACCTGGACAATCCCCAGGAGCCCCGGCCGGAGCGAGCGCCGGCCCCG      60
B                   TGGGATGCCATCGACCTGGACAATCCCCAGGAGCCCCGGCCGGAGCGAGCGCCGGCCCCG      60
AD                  TGGGATGCCATCGACCTGGACAATCCCCAGGAGCCCCGGCCGGAGCGAGCGCCGGCCCCG      60
                    ************************************************************

NM_001009402.2      TCGCCGACCGCCCGGCACGACCAGCCCTCCCTCCTGAACACCCCAGCCACGTGCCAGCGG      120
B                   TCGCCGACCGCCCGGCACGACCAGCCCTCCCTCCTGAACACCCCAGCCACGTGCCAGCGG      120
AD                  TCGCCGACCGCCCGGCACGACCAGCCCTCCCTCCTGAACACCCCAGCCACGTGCCAGCGG      120
                    ************************************************************

NM_001009402.2      TTGCCGAGCCGTCAGGGGAAACCTTCGGTGACCCTAGCTTTGGGCTGGAATATCCCCGCG      180
B                   TTGCCGAGCCGTCGGGGGAAACCTTCGGTGACCCTAGCTTTGGGCTGGAATATCCCCGCG      180
AD                  TTGCCGAGCCGTCAGGGGAAACCTTCGGTGACCCTAGCTTTGGGCTGGAATATCCCCGCG      180
                    ************* **********************************************

NM_001009402.2      TTCCCAGAAACGGAGACTCGGAGTTCGAAGCAGGGTGAACGGACATGGCGGGCTGGATCC      240
B                   TTCCCAGAAACGGAGACTCGGAGTTCGAAGCAGGGTGAACGGACATGGCGGGCTGGATCC      240
AD                  TTCCCAGAAACGGAGACTCGGAGTTCGAAGCAGGGTGAACGGACATGGCGGGCTGGATCC      240
                    ************************************************************

NM_001009402.2      AGGCCCAGCAGCTGCAGGGAGATGCCCTGCGCCAGATGCAGGTGCTATACGGGCAGCACT      300
B                   AGGCCCAGCAGCTGCAGGGAGATGCCCTGCGCCAGATGCAGGTGCTATACGGGCAGCACT      300
AD                  AGGCCCAGCAGCTGCAGGGAGATGCCCTGCGCCAGATGCAGGTGCTATACGGGCAGCACT      300
                    ************************************************************

NM_001009402.2      TCCCCATCGAGGTCCGGCATTACTTGGCGCAGTGGATTGAGAGCCAGCCGTGGGATGCCA      360
B                   TCCCCATCGAGGTCCGGCATTACTTGGCGCAGTGGATTGAGAGCCAGCCGTGGGATGCCA      360
AD                  TCCCCATCGAGGTCCGGCATTACTTGGCGCAGTGGATTGAGAGCCAGCCGTGGGATGCCA      360
                    ************************************************************

NM_001009402.2      TCGACCTGGACAATCCCCAGGACCGGGCCCAGGTCACCCAGCTCCTGGAGGGCCTGGTGC      420
B                   TCGACCTGGACAATCCCCAGGACCGGGCCCAGGTCACCCAGCTCCTGGAGGGCCTGGTGC      420
AD                  TCGACCTGGACAATCCCCAGGACCGGGCCCAGGTCACCCAGCTCCTGGAGGGCCTGGTGC      420
                    ************************************************************

NM_001009402.2      AGGAGTTGCAAAAGAAGGCAGAGCACCAAGTCGGGGAAGACGGGTTCCTACTGAAGATCA      480
B                   AGGAGTTGCAAAAGAAGGCAGAGCACCAAGTCGGGGAAGACGGGTTCCTACTGAAGATCA      480
AD                  AGGAGTTGCAAAAGAAGGCAGAGCACCAAGTCGGGGAAGACGGGTTCCTACTGAAGATCA      480
                    ************************************************************

NM_001009402.2      AGCTGGGGCACTACGTCCACGTCAGCTCCAGAACACGTACGACCGCTGCCCCATGGAGCT      540
B                   AGCTGGGGCACTACGTCCACGTCAGCTCCAGAACACGTACGACCGCTGCCCCATGGAGCT      540
AD                  AGCTGGGGCACTACGTCCACGTCAGCTCCAGAACACGTACGACCGCTGCCCCATGGAGCT      540
                    ************************************************************

NM_001009402.2      GGTTGCGCTGCATTCGCCACATTCTGTACAATGAACAGAGGCTGGTCCGAGAAGCCACCA      600
B                   GGTTGCGCTGCATTCGCCACATTCTGTACAATGAACAGAGGCTGGTCCGAGAAGCCACCA      600
AD                  GGTTGCGCTGCATTCGCCACATTCTGTACAATGAACAGAGGCTGGTCCGAGAAGCCACCA      600
                    ************************************************************

NM_001009402.2      ATGGTAATTCTTCTGCTGGGATCCTGGTTGATGCCATGTCCCAG   644
B                   ATGGTAATTCTTCTGCTGGGATCCTGGTTGATGCCATGTCCCAG   644
AD                  ATGGTAATTCTTCTGCTGGGATCCTGGTTGATGCCATGTCCCAG   644
                    ********************************************
```

Figure S5. Representative DNA sequence alignment for a segment of CDS of *STAT5A* gene (644-bp) among Barki and Aboudeleik lambs and reference sequence available in the GenBank gb|NM_001009402.2|. B= Barki and AD= Aboudeleik. The asterisks represent the similarity.

```
XR_006061143.1      TTAAGACTGAGGGAAGTGGGTGCTGGGAGCCCCACGCCTTCTTTGAGGGCCCGGGAACTG      60
B                   TTAAGACTGAGGGAAGTGGGTGCTGGGAGCCCCACGCCTTCTTTGAGGGCCCGGGAACTG      60
AD                  TTAAGACTGAGGGAAGTGGGTGCTGGGAGCCCCACGCCTTCTTTGAGGGCCCGGGAACTG      60
                    ************************************************************

XR_006061143.1      GAGCTGGGTCAAGCAGTCTCTGAGGTTGGGTTTCCCCAACCAGCCAGCCAGGCAGGCAGA      120
B                   GAGCTGGGTCAAGCAGTCTCTGAGGTTGGGTTTCCCCAACCAGCCAGCCAGGCAGGCAGA      120
AD                  GAGCTGGGTCAAGCAGTCTCTGAGGTTGGGTTTCCCCAACCAGCCAGCCAGGCAGGCAGA      120
                    ************************************************************

XR_006061143.1      AAGGAGACGGTGTCTCAGTGAGCACTTGGCCTGTCTCCCCATGGAGCCCCCCTGCTTCTA      180
B                   AAGGAGACGGTGTCTCAGTGAGCACTTGGCCTGTCTCCCCATGGAGCCCCCCTGCTTCTA      180
AD                  AAGGAGACGGTGTCTCAGTGAGCACTTGGCCTGTCTCCCCATGGAGCCCCCCTGCTTCTA      180
                    ************************************************************

XR_006061143.1      TGCCTTCTCTAAAACCTCCATCAGCCATCTACTTAGTATCAGATGGGGCTTGGGAAGCAG      240
B                   TGCCTTCTCTAAAACCTCCATCAGCCATCTACTTAGTATCAGATGGGGCTTGGGGAGCAG      240
AD                  TGCCTTCTCTAAAACCTCCATCAGCCATCTACTTAGTATCAGATGGGGCTTGGGAAGCAG      240
                    ****************************************************** *****

XR_006061143.1      GCCAGGGTCAGCCTGTGGGCCCAGGGGAAACCCAGGACCTGGAGAATTCAGCTGGGCCCA      300
B                   GCCAGGGTCAGCCTGTGGGCCCAGGGGAAACCCAGGACCTGGAGAATTCAGCTGGGCCCA      300
AD                  GCCAGGGTCAGCCTGTGGGCCCAGGGGAAACCCAGGACCTGGAGAATTCAGCTGGGCCCA      300
                    ************************************************************

XR_006061143.1      GCTTCCCATGTTCCTGATGCCTTTGAGACGTGGCCAGGACAGGACACCAGCGGAGGGGAA      360
B                   GCTTCCCATGTTCCTGATGCCTTTGAGACGTGGCCAGGACAGGACACCAGCGGAGGGGAA      360
AD                  GCTTCCCATGTTCCTGATGCCTTTGAGACGTGGCCAGGACAGGACACCAGCGGAGGGGAA      360
                    ************************************************************

XR_006061143.1      TGAACTGTGGCCTGAGGGCAGAAGTTGCAGGGAGGAACTGGAAGAACTTTGCCCTGGTTC      420
B                   TGAACTGTGGCCTGAGGGCAGAAGTTGCAGGGAGGAACTGGAAGAACTTTGCCCTGGTTC      420
AD                  TGAACTGTGGCCTGAGGGCAGAAGTTGCAGGGAGGAACTGGAAGAACTTTGCCCTGGTTC      420
                    ************************************************************

XR_006061143.1      CCCTCTTACGAGACGCAAGTACTCGAGAAAGGCACCCTGCCCAGCCTGAAGAAGTTCACC      480
B                   CCCTCTTACGAGATGCAAGTACTCGAGAAAGGCACCCTGCCCAGCCTGAAGAAGTTCACC      480
AD                  CCCTCTTACGAGACGCAAGTACTCGAGAAAGGCACCCTGCCCAGCCTGAAGAAGTTCACC      480
                    ************* **********************************************

XR_006061143.1      TGAGGCACTTCACAGG   496
B                   TGAGGCACTTCACAGG   496
AD                  TGAGGCACTTCACAGG   496
                    ****************
```

Figure S6. Representative DNA sequence alignment for a segment of CDS of *TRPV1* gene (496-bp) among Barki and Aboudeleik lambs and reference sequence available in the GenBank gb|XR_006061143.1|. B= Barki and AD= Aboudeleik. The asterisks represent the similarity.

```
XM_004018854.5      GTGCTTCGCCTTATATAAAGTGATTGGGGGCACACAGCCGTTCTCTCGAGTCACCCCCGC      60
B                   GTGCTTCGCCTTATATAAAGTGATTGGGGGCACACAGCCGTTCTCTCGAGTCACCCCCGC      60
AD                  GTGCTTCGCCTTATATAAAGTGATTGGGGGCACACAGCCGTTCTCTCGAGTCACCCCCGC      60
                    ************************************************************

XM_004018854.5      GCAGCCTTCTTGTGGCGAGTGAGCCCGAGGAAGCCATTGCCTGCTCGGTGTTCTCTAATA      120
B                   GCAGCCTTCTTGTGGCGAGTGAGCCCGAGGAAGCCATTGCCTGCTCGGTGTTCTCTAGTA      120
AD                  GCAGCCTTCTTGTGGCGAGTGAGCCCGAGGAAGCCATTGCCTGCTCGGTGTTCTCTAATA      120
                    ********************************************************* **

XM_004018854.5      CCTGTGATTGTTATCCTTGAAGATGCCTGAGGAAGTGCACCATGGAGAGGAGGAGGTGGA      180
B                   CCTGTGATTGTTATCCTTGAAGATGCCTGAGGAAGTGCACCATGGAGAGGAGGAGGTGGA      180
AD                  CCTGTGATTGTTATCCTTGAAGATGCCTGAGGAAGTGCACCATGGAGAGGAGGAGGTGGA      180
                    ************************************************************

XM_004018854.5      GACATTTGCCTTCCAGGCAGAAATTGCCCAACTCATGTCTCTTATTATCAACACCTTCTA      240
B                   GACATTTGCCTTCCAGGCAGAAATTGCCCAACTCATGTCTCTTATTATCAATACCTTCTA      240
AD                  GACATTTGCCTTCCAGGCAGAAATTGCCCAACTCATGTCTCTTATTATCAACACCTTCTA      240
                    *************************************************** ********

XM_004018854.5      TTCCAACAAGGAAATTTTTCTTCGGGAGTTGATCAGTAATGCTTCTGATGCCTTGGACAA      300
B                   TTCCAACAAGGAAATTTTTCTTCGGGAGTTGATCAGTAATGCTTCTGATGCCTTGGACAA      300
AD                  TTCCAACAAGGAAATTTTTCTTCGGGAGTTGATCAGTAATGCTTCTGATGCCTTGGACAA      300
                    ************************************************************

XM_004018854.5      GATTCGCTATGAAAGCCTGACAGACCCTTCTAAATTGGACAGTGGTAAAGAGCTGAAAAT      360
B                   GATTCGCTATGAAAGCCTGACAGACCCTTCTAAATTGGACAGTGGTAAAGAGCTGAAAAT      360
AD                  GATTCGCTATGAAAGCCTGACAGACCCTTCTAAATTGGACAGTGGTAAAGAGCTGAAAAT      360
                    ************************************************************

XM_004018854.5      TGACATCATCCCGAATCCCCAGGAGCGTACTCTGACTCTGGTAGACACGGGCATTGGCAT      420
B                   TGACATCATCCCGAATCCCCAGGAGCGTACTCTGACTCTGGTAGACACGGGCATTGGCAT      420
AD                  TGACATCATCCCGAATCCCCAGGAGCGTACTCTGACTCTGGTAGACACGGGCATTGGCAT      420
                    ************************************************************

XM_004018854.5      GACCAAAGCTGATCTTGTAAATAATTTGGGAACCATTGCCAAGTCTGGCACTAAAGCATT      480
B                   GACCAAAGCTGATCTTGTAAATAATTTGGGAACCATTGCCAAGTCTGGCACTAAAGCGTT      480
AD                  GACCAAAGCTGATCTTGTAAATAATTTGGGAACCATTGCCAAGTCTGGCACTAAAGCATT      480
                    ********************************************************* **

XM_004018854.5      TATGGAGGCTCTTCAGGCTGGTGCAGACATCTCCATGATTGGGCAG 526
B                   TATGGAGGCTCTTCAGGCTGGTGCAGACATCTCCATGATTGGGCAG 526
AD                  TATGGAGGCTCTTCAGGCTGGTGCAGACATCTCCATGATTGGGCAG 526
                    **********************************************
```

Figure S7. Representative DNA sequence alignment for a segment of CDS of *HSP90AB1* gene (526-bp) among Barki and Aboudeleik lambs and reference sequence available in the GenBank gb|XM_004018854.5|. B= Barki and AD= Aboudeleik. The asterisks represent the similarity.

```
XM_027977443.2      ACTGCGGAGCCTAGGGCGACGGCAGAGCAGGATGGAGATCCCAGTGCCTGTGCAGCCGTC      60
B                   ACTGCGGAGCCTAGGGCGACGGCAGAGCAGGATGGAGATCCCAGTGCCTGTGCAGCCGTC      60
AD                  ACTGCGGAGCCTAGGGCGACGGCAGAGCAGGATGGAGATCCCAGTGCCTGTGCAGCCGTC      60
                    ************************************************************

XM_027977443.2      TTGGCTGCGCCGCGCCTCGGCCCCTTTGCCTGGGCTGTCGGCTCCCGGGCGCCTCTTCGA      120
B                   TTGGCTGCGCCGCGCCTCGGCCCCTTTGCCTGGGCTGTCGGCTCCCGGGCGCCTCTTCGA      120
AD                  TTGGCTGCGCCGCGCCTCGGCCCCTTTGCCTGGGCTGTCGGCTCCCGGGCGCCTCTTCGA      120
                    ************************************************************

XM_027977443.2      CCAGCGCTTCGGCGAGGGGCTGCTGGAGGCCGAGCTGGCTGCGCTCTGCCCTGCCGCGCT      180
B                   CCAGCGCTTCGGCGAGGGGCTGCTGGAGGCCGAGCTGGCTGCGCTCTGCCCTGCCGCGCT      180
AD                  CCAGCGCTTCGGCGAGGGGCTGCTGGAGGCCGAGTTGGCTGCGCTCTGCCCTGCCGCGCT      180
                    ********************************** *************************

XM_027977443.2      GGCCCCCTACTACCTGCGCGCACCCAGCGTGGCGCTGCCTACCGCCCAGGTATCTACCGA      240
B                   GGCCCCCTACTACCTGCGCGCACCCAGCGTGGCGCTGCCTACCGCCCAGGTATCTACCGA      240
AD                  GGCCCCCTACTACCTGCGCGCACCCAGCGTGGCGCTGCCTACCGCCCAGGTATCTACCGA      240
                    ************************************************************

XM_027977443.2      CCCCGGGCATTTCTCGGTGCTGCTGGATGTGAAACACTTCTCACCCGAGGAAATTGCCGT      300
B                   CCCCGGGCATTTCTCGGTGCTGCTGGATGTGAAACACTTCTCACCCGAGGAAATTGCCGT      300
AD                  CCCCGGGCATTTCTCGGTGCTGCTGGATGTGAAACACTTCTCACCCGAGGAAATTGCCGT      300
                    ************************************************************

XM_027977443.2      CAAGGTGGTTGGTGACCACGTGGAAGTTCATGCGCGTCACGAGGAGCGCCCGGATGAGCA      360
B                   CAAGGTGGTTGGTGACCACGTGGAAGTTCATGCGCGTCACGAGGAGCGCCCGGATGAGCA      360
AD                  CAAGGTGGTTGGTGACCACGTGGAAGTTCATGCGCGTCACGAGGAGCGCCCGGATGAGCA      360
                    ************************************************************

XM_027977443.2      CGGATACATTGCGCGCGAGTTCCACCGCCGCTACCGCTTGCCGCCTGGTGTGGACCCTGC      420
B                   CGGATACATTGCGCGCGAGTTCCACCGCCGCTACCGCTTGCCGCCTGGTGTGGACCCTGC      420
AD                  CGGATACATTGCGCGCGAGTTCCACCGCCGCTACCGCTTGCCGCCTGGTGTGGACCCTGC      420
                    ************************************************************

XM_027977443.2      GGCCGTGACGTCTGCGCTGTCCCCTGAGGGCGTCCTTTCCATCCAGGCCGCACCTGCGCC      480
B                   GGCCGTGACGTCTGCGCTGTCCCCTGAGGGCGTCCTTTCCATCCAGGCCGCACCTGCGCC      480
AD                  GGCCGTGACGTCTGCGCTGTCCCCTGAGGGCGTCCTTTCCATCCAGGCCGCACCTGCGCC      480
                    ************************************************************

XM_027977443.2      GGCCCAGGCCCCACTGCAGTCGCCGCCCGGGGCGGCTGCCAAGTAGGGGTCAGGGCACAC      540
B                   GGCCCAGGCCCCACTGCAGTCGCCGCCCGGGGCGGCTGCCAAGTAGGGGTCAGGGCACAC      540
AD                  GGCCCAGGCCCCACTGCAGTCGCCGCCCGGGGCGGCTGCCAAGTAGGGGTCAGGGCACAC      540
                    ************************************************************

XM_027977443.2      CTGAACCCTGGGAGCCGCCTCAGCGCCCCCGCCCTTTCAAAGCCGACCTGACTCCGCCCA      600
B                   CTGAACCCTGGGAGCCGCCTCAGCGCCCCCGCCCTTTCAAAGCCGACCTGACTCCGCCCA      600
AD                  CTGAACCCTGGGAGCCGCCTCAGCGCCCCCGCCCTTTCAAAGCCGACCTGACTCCGCCCA      600
                    ************************************************************

XM_027977443.2      GCCAGGTGTCCCCAGCGCGCGCCAAGACTA        630
B                   GCCAGGTGTCCCCAGCGCGCGCCAAGACTA        630
AD                  GCCAGGTGTCCCCAGCGCGCGCCAAGACTA        630
                    ******************************
```

Figure S8. Representative DNA sequence alignment for a segment of CDS of *HSPB6* gene (630-bp) among Barki and Aboudeleik lambs and reference sequence available in the GenBank gb|XM_027977443.2|. B= Barki and AD= Aboudeleik. The asterisks represent the similarity.

```
XM_027973370.2      GGCCTCTCTTTGCGGTTGCTACCTCCTCCTCTCCCGTCTCGCCCGGTATGGATCTGCCCG      60
B                   GGCCTCTCTTTGCGGTTGCTACCTCCTCCTCTCCCGTCTCGCCCGGTATGGATCTGCCCG      60
AD                  GGCCTCTCTTTGCGGTTGCTACCTCCTCCTCTCCCGTCTCGCCCGGTATGGATCTGCCCG      60
                    ************************************************************

XM_027973370.2      TGGGCCCGGGCGCGGCGGGGCCCAGCAACGTCCCGGCCTTCCTGACCAAGCTGTGGACTC      120
B                   TGGGCCCGGGCGCGGCGGGGCCCAGCAACGTCCCGGCCTTCCTGACCAAGCTGTGGACTC      120
AD                  TGGGCCCGGGCGCGGCGGGGCCCAGCAACGTCCCGGCCTTCCTGACCAAGCTGTGGACTC      120
                    ************************************************************

XM_027973370.2      TCGTGAGCGACCCGGACACGGACGCGCTCATCTGCTGGAGCCCGAGCGGGAACAGCTTCC      180
B                   TCGTGAGCGACCCGGACACGGACGCGCTCATCTGCTGGAGCCCGAGCGGGAACAGCTTCC      180
AD                  TCGTGAGCGACCCGGACACGGACGCGCTCATCTGCTGGAGCCCGAGCGGAAACAGCTTCC      180
                    ************************************************* **********

XM_027973370.2      ACGTGCTGGACCAGGGCCAGTTTGCCAAGGAGGTGCTGCCCAAGTACTTCAAGCACAGCA      240
B                   ACGTGCTGGACCAGGGCCAGTTTGCCAAGGAGGTGCTGCCCAAGTACTTCAAGCACAGCA      240
AD                  ACGTGCTGGACCAGGGCCAGTTTGCCAAGGAGGTGCTGCCCAAGTACTTCAAGCACAGCA      240
                    ************************************************************

XM_027973370.2      ACATGGCTAGCTTCGTGCGGCAGCTCAACATGTATGGCTTCCGGAAGGTGGTCCACATCG      300
B                   ACATGGCTAGCTTCGTGCGGCAGCTCAACATGTATGGCTTCCAGAAGGTGGTCCACATCG      300
AD                  ACATGGCTAGCTTCGTGCGGCAGCTCAACATGTATGGCTTCCGGAAGGTGGTCCACATCG      300
                    ****************************************** *****************

XM_027973370.2      AGCAGGGTGGCCTGGTCAAGCCAGAGAGGGACGACACCGAGTTCCAGCACCCGTGCTTCC      360
B                   AGCAGGGTGGCCTGGTCAAGCCAGAGAGGGACGACACCGAGTTCCAGCACCCGTGCTTCC      360
AD                  AGCAGGGTGGCCTGGTCAAGCCAGAGAGGGACGACACCGAGTTCCAGCACCCGTGCTTCC      360
                    ************************************************************

XM_027973370.2      TGCGAGGCCAGGAGCAGCTCCTCGAGAACATCAAGAGGAAAGTGACCAGCGTGTCCACTC      420
B                   TGCGAGGCCAGGAGCAGCTCCTCGAGAACATCAAGAGGAAAGTGACCAGCGTGTCCACTC      420
AD                  TGCGAGGCCAGGAGCAGCTCCTCGAGAACATCAAGAGGAAAGTGACCAGTGTGTCCACTC      420
                    ************************************************* **********

XM_027973370.2      AGCTGATGAAGGGGAAGCAGGAGAGCATGGACTCCAAGCTGCTGGCCATGAAGCATGAGA      480
B                   AGCTGATGAAGGGGAAGCAGGAGAGCATGGACTCCAAGCTGCTGGCCATGAAGCATGAGA      480
AD                  AGCTGATGAAGGGGAAGCAGGAGAGCATGGACTCCAAGCTGCTGGCCATGAAGCATGAGA      480
                    ************************************************************
```

Figure S9. Representative DNA sequence alignment for a segment of CDS of *HSF1* gene (480-bp) among Barki and Aboudeleik lambs and reference sequence available in the GenBank gb|XM_027973370.2|. B= Barki and AD= Aboudeleik. The asterisks represent the similarity.

```
XM_042238340.1      GAATAACGGAGGCAGAGCCCCTTCATTAACTGCTAAAAACTCTACTGCACGAATGTGAAC      60
B                   GAATAACGGAGGCAGAGCCCCTTCATTAACTGCTAAAAACTCTACTGCACGAATGTGAAC      60
AD                  GAATAACGGAGGCAGAGCCCCTTCATTAACTGCTAAAAACTCTACTGCACGAATGTGAAC      60
                    ************************************************************

XM_042238340.1      GATCCCACGCCACGGCCCTCTACCTGAGAGGTTCAGCGAGGGTGTGCGTCTCCACCGAGG      120
B                   GATCCCACGCCACGGCCCTCTACCTGAGAGGTTCAGCGAGGGTGTGCGTCTCCACCGAGG      120
AD                  GATCCCACGCCACGGCCCTCTACCTGAGAGGTTCAGCGAGGGTGTGCGTCTCCACCGAGG      120
                    ************************************************************

XM_042238340.1      AGATTGGCCAAGATATAATTCAGAGCCTGTCCTACCCGAGCCCCAGCGCCTTCACCATCA      180
B                   AGATTGGCCAAGATATAATTCAGAGCCTGTCCTACCCGAGCCCCAGCGCCTTCACCGTCA      180
AD                  AGATTGGCCAAGATATAATTCAGAGCCTGTCCTACCCGAGCCCCAGCGCCTTCACCATCA      180
                    ******************************************************** ***

XM_042238340.1      CGCGGGATTTGGGGACACCCAGAACCCAATATTGGGACTCAGGGACGGGCCAGGGTGCGC      240
B                   CGCGGGATTTGGGGACACCCAGAACCCAATATTGGGACTCAGGGACGGGCCAGGGTGCGC      240
AD                  CGCGGGATTTGGGGACACCCAGAACCCAATATTGGGACTCAGGGACGGGCCAGGGTGCGC      240
                    ************************************************************

XM_042238340.1      TCCCGAGGCCGATCCAAACGGTCACCCCCACTGGCTGGGACCCTGACACTCCGGCGAAGG      300
B                   TCCCGAGGCCGATCCAAACGGTCACCCCCACTGGCTGGGACCCTGACACTCCGGCGAAGG      300
AD                  TCCCGAGGCCGATCCAAACGGTCACCCCCACTGGCTGGGACCCTGACACTCCGGCGAAGG      300
                    ************************************************************

XM_042238340.1      ACCAAGACTCAATGCCGCCGGAGAGAAGCGAACTGCTTCAGACTGACTCGCCGCAGACCA      360
B                   ACCAAGACTCAATGCCGCCGGAGAGAAGCGAACTGCTTCAGACTGACTCGCCGCAGACCA      360
AD                  ACCAAGACTCAATGCCGCCGGAGAGAAGCGAACTGTTTCAGACTGACTCGCCGCAGACCA      360
                    *********************************** ************************

XM_042238340.1      ACGGAAGAAGCGGCCCGGGCGGACCCACCAAACCCACACCCTCAGAAACCACCACTGCAG      420
B                   ACGGAAGAAGCGGCCCGGGCGGACCCACCAAACCCACACCCTCAGAAACCACCACTGCAG      420
AD                  ACGGAAGAAGCGGCCCGGGCGGACCCACCAAACCCACACCCTCAGAAACCACCACTGCAG      420
                    ************************************************************

XM_042238340.1      AGGGTGTCTGTGGCCTCGGACTCCCATAAGCCACCGATGCTGTAAAAAGCCTACCCTGCT      480
B                   AGGGTGTCTGTGGCCTCGGACTCCCATAAGCCACCGATGCTGTAAAAAGCCTACCCTGCT      480
AD                  AGGGTGTCTGTGGCCTCGGACTCCCATAAGCCACCGCTGCTGTAAAAAGCCTACCCTGCT      480
                    ************************************ ***********************

XM_042238340.1      CTTTGAAGGGCAGCAATTTAAACCTACCAGAGCAAAGTGTCTGCAACCACCGACCAATTG      540
B                   CTTTGAAGGGCAGCAATTTAAACCTACCAGAGCAAAGTGTCTGCAACCACCGACCAATTG      540
AD                  CTTTGAAGGGTAGCAATTTAAACCTACCAGAGCAAAGTGTCTGCAACCACCGACCAATTG      540
                    ********** *************************************************
```

Figure S10. Representative DNA sequence alignment for a segment of CDS of *ST1P1* gene (540-bp) among Barki and Aboudeleik lambs and reference sequence available in the GenBank gb|XM_042238340.1|. B= Barki and AD= Aboudeleik. The asterisks represent the similarity.

```
KP325220.1      CCTGCGTGGTGCATGGAAGTGATCTGAAGGACATGACTCCCGAGCAACTGGATGACATTT 60
B               CCTGCGTGGTGCATGGAAGTGATCTGAAGGACATGACTCCCGAGCAACTGGATGACATTT 60
AD              CCTGCGTGGTGCATGGAAGTGATCTGAAGGACATGACTCCCGAGCAGCTGGATGACATTT 60
                ********************************************* **************

KP325220.1      TGAAGTACCACACGGAGATTGTGTTCGCCAGGACCTCTCCGCAGCAGAAGCTCATCATTG 120
B               TGAAGTACCACACGGAGATTGTGTTCGCCAGGACCTCTCCGCAGCAGAAGCTCATCATTG 120
AD              TGAAGTACCACACGGAGATTGTGTTCGCCAGGACCTCTCCGCAGCAGAAGCTCATCATTG 120
                ************************************************************

KP325220.1      TGGAAGGCTGCCAGAGACAGGTCGGCACACAGCTGGGGCGGGCTCTTTCAGACGCTGCTG 180
B               TGGAAGGCTGCCAGAGACAGGTTGGCACACAGCTGGGGCGGGCTCTTTCAGACGCTGCTG 180
AD              TGGAAGGCTGCCAGAGACAGGTCGGCACACAGCTGGGGCGGGCTCTTTCAGACGCTGCTG 180
                ********************* **************************************

KP325220.1      GCCTGGCCTCAGTGTAGACAAAAAGCAAGGCAACAGTGCTGGAAACCTGAGCCAGCTAGC 240
B               GCCTGGCCTCAGTGTAGACAAAAAGCAAGGCAACAGTGCTGGAAACCTGAGCCAGCTAGC 240
AD              GCCTGGCCTCAGTGTAGACAAAAAGCAAGGCAACAGTGCTGGAAACCTGAGCCAGCTAGC 240
                ************************************************************

KP325220.1      GCCTCTCTGTCTCAGTACACATGGGCCAGTAAGAAGCTCCAGGCGTAGAGGATGGTGAC  299
B               GCCTCTCTGTCTCAGTACACATGGGCCAGTAAGAAGCTCCAGGCGTAGAGGATGGTGAC  299
AD              GCCTCTCTGTCTCAGTACACATGGGCCAGTAAGAAGCTCCAGGCGTAGAGGATGGTGAC  299
                ***********************************************************
```

Figure S11. Representative DNA sequence alignment for a segment of CDS of *ATP1A1* gene (299-bp) among Barki and Aboudeleik lambs and reference sequence available in the GenBank gb|KP325220.1|. B= Barki and AD= Aboudeleik. The asterisks represent the similarity.

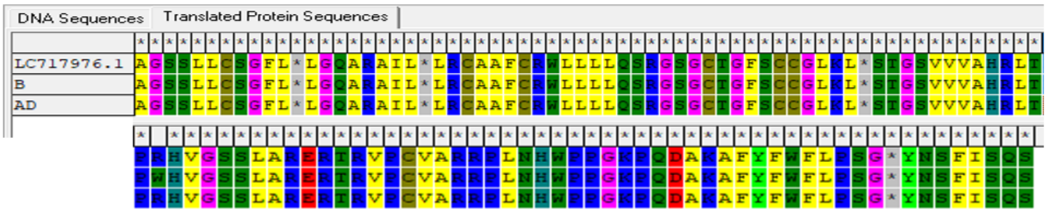

Figure S12. Representative amino acid sequence alignment of *CAST* gene (354-bp) among Barki and Aboudeleik lambs and reference sequence available in the GenBank gb|LC717976.1|. B= Barki and AD= Aboudeleik. The asterisks represent the similarity.

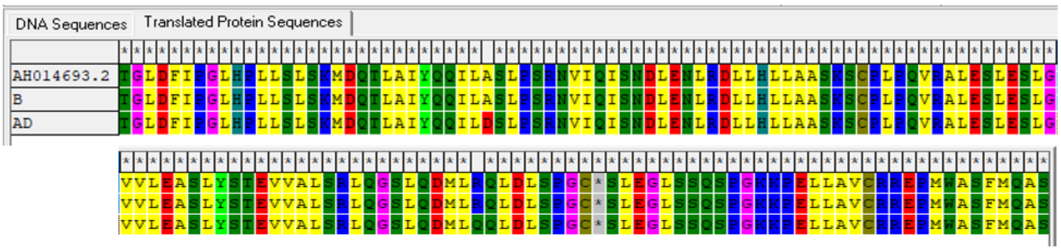

Figure S13. Representative amino acid sequence alignment of *LEP* gene (432-bp) among Barki and Aboudeleik lambs and reference sequence available in the GenBank gb|AH014693.2|. B= Barki and AD= Aboudeleik. The asterisks represent the similarity.

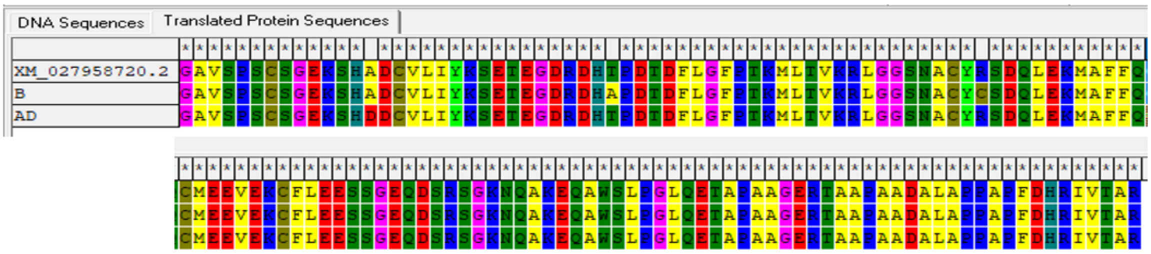

Figure S14. Representative amino acid sequence alignment of *MYLK4* gene (416-bp) among Barki and Aboudeleik lambs and reference sequence available in the GenBank gb|XM_027958720.2|. B= Barki and AD= Aboudeleik. The asterisks represent the similarity.

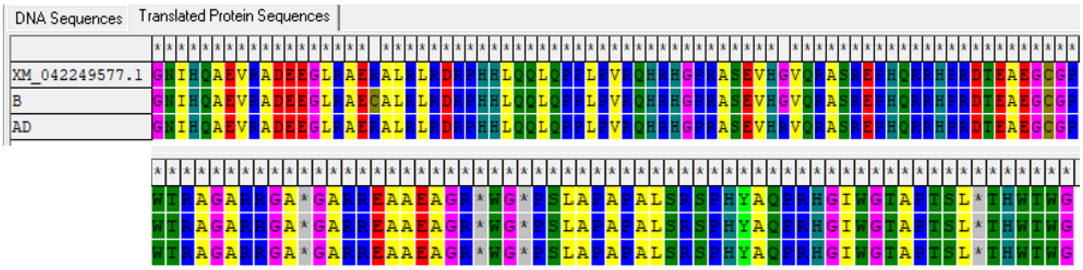

Figure S15. Representative amino acid sequence alignment of *MEF2B* gene (424-bp) among Barki and Aboudeleik lambs and reference sequence available in the GenBank gb|XM_042249577.1|. B= Barki and AD= Aboudeleik. The asterisks represent the similarity.

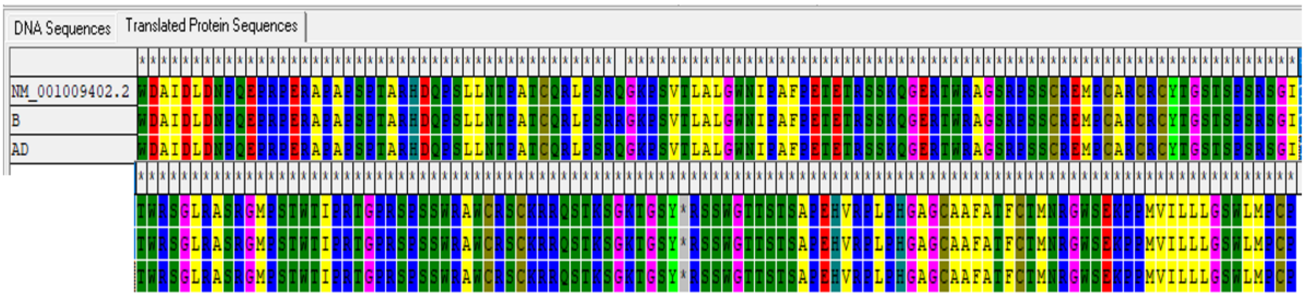

Figure S16. Representative amino acid sequence alignment of *STAT5A* gene (644-bp) among Barki and Aboudeleik lambs and reference sequence available in the GenBank gb|NM_001009402.2|. B= Barki and AD= Aboudeleik. The asterisks represent the similarity.

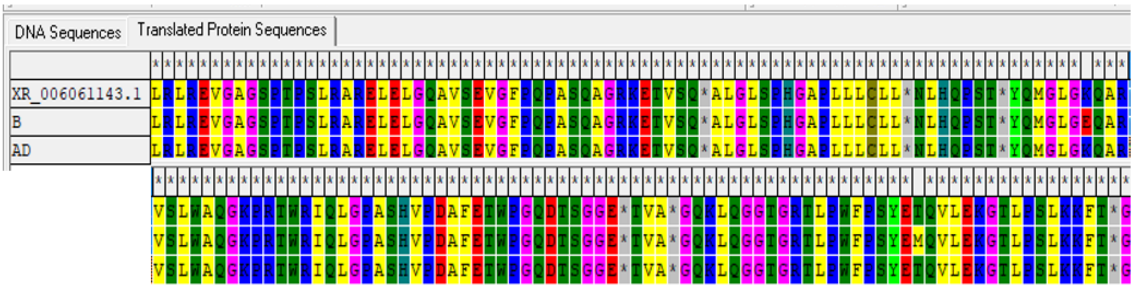

Figure S17. Representative amino acid sequence alignment of *TRPV1* gene (496-bp) among Barki and Aboudeleik lambs and reference sequence available in the GenBank gb|XR_006061143.1|. B= Barki and AD= Aboudeleik. The asterisks represent the similarity.

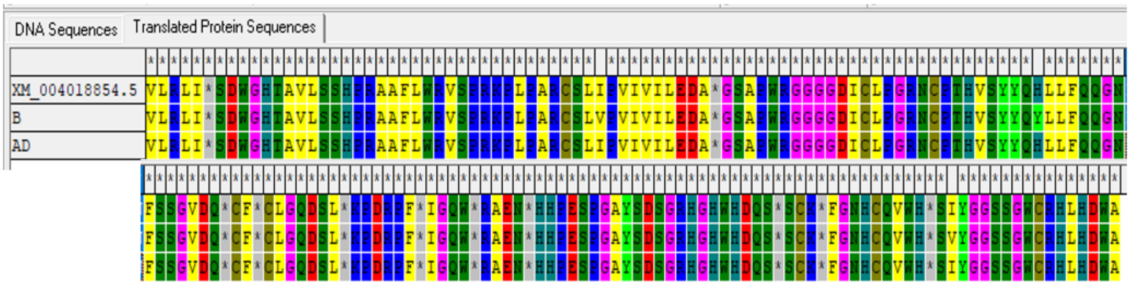

Figure S18. Representative amino acid sequence alignment of *HSP90AB1* gene (526-bp) among Barki and Aboudeleik lambs and reference sequence available in the GenBank gb|XM_004018854.5|. B= Barki and AD= Aboudeleik. The asterisks represent the similarity.

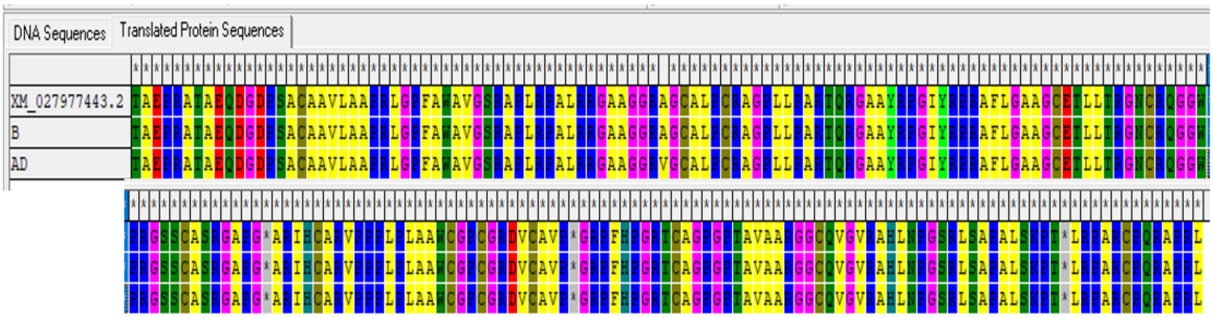

Figure S19. Representative amino acid sequence alignment of *HSPB6* gene (630-bp) among Barki and Aboudeleik lambs and reference sequence available in the GenBank gb|XM_027977443.2|. B= Barki and AD= Aboudeleik. The asterisks represent the similarity.

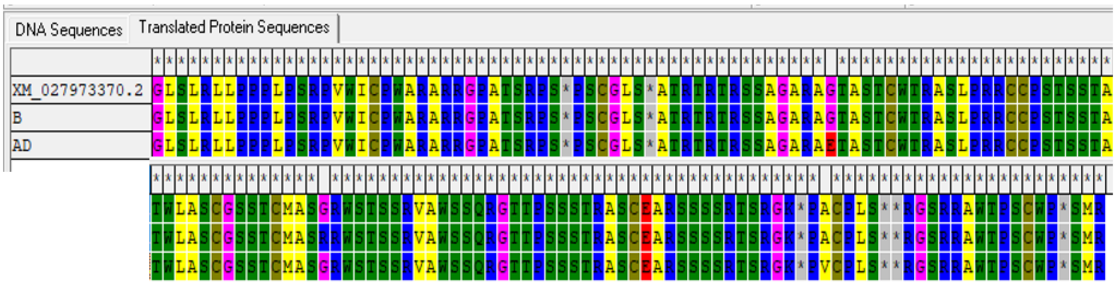

Figure S20. Representative amino acid sequence alignment of *HSF1* gene (480-bp) among Barki and Aboudeleik lambs and reference sequence available in the GenBank gb|XM_027973370.2|. B= Barki and AD= Aboudeleik. The asterisks represent the similarity.

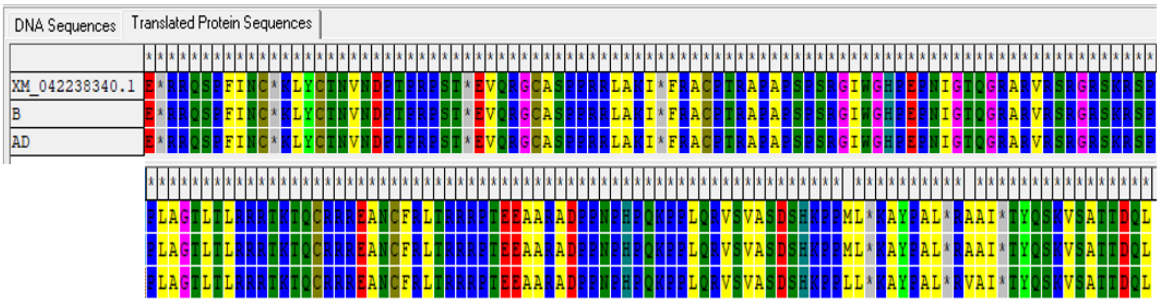

Figure S21. Representative amino acid sequence alignment of *ST1P1* gene (540-bp) among Barki and Aboudeleik lambs and reference sequence available in the GenBank gb|XM_042238340.1|. B= Barki and AD= Aboudeleik. The asterisks represent the similarity.

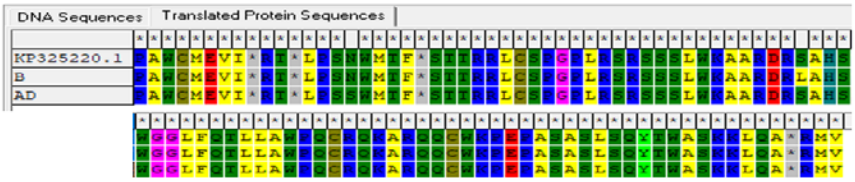

Figure S22. Representative amino acid sequence alignment of *ATP1A1* gene (299-bp) among Barki and Aboudeleik lambs and reference sequence available in the GenBank gb|KP325220.1|. B= Barki and AD= Aboudeleik. The asterisks represent the similarity.
